# Dapsone Azo-Linked with Two Mesalazine Moieties Is a “Me-Better” Alternative to Sulfasalazine

**DOI:** 10.3390/pharmaceutics14030684

**Published:** 2022-03-21

**Authors:** Changyu Kang, Jaejeong Kim, Sanghyun Ju, Sohee Park, Jin-Wook Yoo, In-Soo Yoon, Min-Soo Kim, Yunjin Jung

**Affiliations:** College of Pharmacy, Pusan National University, Busan 46241, Korea; whale10000@naver.com (C.K.); wowjd9669@naver.com (J.K.); jsh141002@naver.com (S.J.); psh7728@pusan.ac.kr (S.P.); jinwook@pusan.ac.kr (J.-W.Y.); insoo.yoon@pusan.ac.kr (I.-S.Y.); minsookim@pusan.ac.kr (M.-S.K.)

**Keywords:** dapsone, mesalazine, colon-specific prodrug, “me-better” drug, inflammatory bowel disease, sulfasalazine, azo bond

## Abstract

Dapsone (DpS) is an antimicrobial and antiprotozoal agent, especially used to treat leprosy. The drug shares a similar mode of action with sulfonamides. Additionally, it possesses anti-inflammatory activity, useful for treating autoimmune diseases. Here, we developed a “me-better” alternative to sulfasalazine (SSZ), a colon-specific prodrug of mesalazine (5-ASA) used as an anti-inflammatory bowel diseases drug; DpS azo-linked with two molecules of 5-ASA (AS-DpS-AS) was designed and synthesized, and its colon specificity and anti-colitic activity were evaluated. AS-DpS-AS was converted to DpS and the two molecules of 5-ASA (up to approximately 87% conversion) within 24 h after incubation in the cecal contents. Compared to SSZ, AS-DpS-AS showed greater efficiency in colonic drug delivery following oral gavage. Simultaneously, AS-DpS-AS substantially limited the systemic absorption of DpS. In a dinitrobenzene sulfonic acid-induced rat colitis model, oral AS-DpS-AS elicited better efficacy against rat colitis than oral SSZ. Moreover, intracolonic treatment with DpS and/or 5-ASA clearly showed that combined treatment with DpS and 5-ASA was more effective against rat colitis than the single treatment with either DpS or 5-ASA. These results suggest that AS-DpS-AS may be a “me-better” drug of SSZ with higher therapeutic efficacy, owing to the combined anti-colitic effects of 5-ASA and DpS.

## 1. Introduction

Inflammatory bowel disease (IBD), comprising of ulcerative colitis (UC) and Crohn’s disease (CD), is a chronic and relapsing inflammatory disorder that occurs mainly in the distal part of the gastrointestinal (GI) tract. The exact etiopathogenesis of IBD remains elusive despite extensive research over the last few decades [1,2]. Pharmacotherapeutics for the treatment of IBD, including aminosalicylates, glucocorticoids, and immunosuppressants, now include biologics, such as anti-TNF-α drugs, aiming at induction and long-term maintenance of remission [3]. However, a considerable percentage of IBD patients are not satisfactorily responsive to IBD therapeutics and/or have to discontinue medication, likely due to ineffectiveness, tolerance, and adverse effects [3,4].

Mesalamine (5-aminosalicylic acid, 5-ASA) is an anti-inflammatory drug that is widely used for the treatment of mild to moderate IBD [5]. Although the therapeutic use of 5-ASA as an anti-IBD drug is limited in patients with mild IBD, probably due to its low anti-inflammatory efficacy, 5-ASA has advantages over other anti-IBD medications in terms of safety and pharmacoeconomics [6,7]. Since 5-ASA itself is extensively absorbed in the upper intestine, causing systemic side effects rather than anti-IBD effects, it is not suitable for therapeutic use against IBD via the oral route. 5-ASA is pharmaceutically formulated or chemically modified to optimize its effectiveness against IBD and minimize toxicological concerns [7]. Pharmaceutical formulations of 5-ASA designed to release the drug from the distal part of the small intestine (the terminal ileum) are achieved by coating the drug with polymers sensitive to pH (representative commercial product: Asacol) or by developing a formulation suitable for time-controlled release (representative commercial product: Pentasa) [8,9]. Whereas 5-ASA is chemically modified for therapeutic and toxicological optimization, leading to the discovery of colon-specific prodrugs of 5-ASA [10]. While remaining intact and less permeable to the epithelial layer during transit in the stomach and small intestine, the prodrugs release active 5-ASA and its carriers in the large intestine [10,11]. Colon-specific prodrugs of 5-ASA that are clinically available for the treatment of IBD are sulfasalazine (SSZ), olsalazine, and balsalazide. There is no clinical difference among these prodrugs, and they are therapeutically equivalent as anti-IBD drugs [12,13,14].

SSZ, a conjugate of 5-ASA and the antimicrobial sulfonamide sulfapyridine linked via an azo bond, cleavable by the microbial enzyme(s) in the large intestine, is a colon-specific prodrug of 5-ASA, currently used for the treatment of mild to moderate IBD, particularly ulcerative colitis [15,16]. Initially, SSZ was developed as an anti-IBD drug, where the antimicrobial effect of sulfapyridine, as anticipated, would combine with the anti-inflammatory effect of the drug 5-ASA to treat IBD. However, many studies have indicated 5-ASA to be an active agent and sulfapyridine as therapeutically inactive against IBD, showing side effects [13]. To improve the toxicological properties of SSZ, olsalazine, and balsalazide, consisting of 5-ASA and aminohippuric acid, respectively, instead of sulfapyridine, were developed as “me-too” drugs for SSZ [7,10,13].

Dapsone (DpS), 4,4-diaminodiphenylsulfone, is an antimicrobial and antiprotozoal agent, particularly used for the treatment of Hansen’s disease (leprosy) caused by mycobacterial infection. As DpS was not envisioned as an antimicrobial agent during its first reported syntheses in 1908 [17], its research as an antimicrobial medicine was initiated approximately 20 years later, facilitated by studies on the synthetic antimicrobial agent, sulfonamides, structurally similar to DpS with a sulfone moiety. In fact, DpS and sulfonamides have similar modes of action (inhibition of folic acid synthesis) as antimicrobial agents [17,18]. In addition, DpS (found by serendipity) has anti-inflammatory activity, is clinically useful for the treatment of autoimmune diseases causing dermatological and cartilage problems such as cutaneous lupus erythematosus, chronic spontaneous urticaria, relapsing polychondritis, dermatitis herpetiformis, and erythema elevatum diutinum [17,19]. Along with its therapeutic usefulness, DpS is inexpensive and is available in various dosage forms. Although DpS elicits side effects, including hematologic effects, which are similar to those of sulfonamides [17,18], it is included in the World Health Organization’s List of Essential Medicines, which consist of all the safest and most effective medicines needed in the health system.

Unlike the carriers of clinically available 5-ASA prodrugs, such as sulfapyridine, DpS, as a complete drug, is effective against experimental colitis [20,21]. It has two aniline moieties that can form azo-conjugation with two molecules of 5-ASA. Based on the pharmacological and chemical features of DpS, the two aniline moieties in DpS were coupled with two molecules of 5-ASA to yield DpS azo-linked to two molecules of 5-ASA (AS-DpS-AS) and AS-DpS-AS, activated to 5-ASA and DpS in large intestine, are expected to exert synergistic anti-colitic effects, thus acting as a codrug or mutual prodrug [22,23,24,25].

AS-DpS-AS was evaluated as an efficacious SSZ alternative (i.e., a “me-better” drug), and its colon specificity and anti-colitic activity were compared to those of SSZ.

## 2. Materials and Methods

### 2.1. Materials

DpS, salicylic acid (SA), sodium nitrite (NaNO_2_), sulfamic acid, and 2,4-dinitrobenzene sulfonic acid (DNBS) were purchased from the Tokyo Chemical Industry Co., Ltd. (Tokyo, Japan). Sulfasalazine (SSZ) was purchased from Sigma-Aldrich Chemical Co. Inc. (St. Louis, MO, USA). Reaction solvents and high-performance liquid chromatography (HPLC) grade solvents were obtained from Junsei Chemical Co. (Tokyo, Japan) and Daejung Chemicals & Metals Co. Ltd. (Gyeonggi-do, Siheung, Korea), respectively. Cytokine-induced neutrophil chemoattractant-3 (CINC-3) enzyme-linked immunosorbent assay (ELISA) kit was purchased from R&D Systems Inc. (Minneapolis, MN, USA). Phosphate buffer saline (pH 7.4) was purchased from Thermo Fisher Scientific (Waltham, MA, USA). All other chemicals used were commercially available reagent-grade reagents. Spots on thin-layer chromatography (TLC) plates (silica gel F_254_s, Merck Millipore, Burlington, MA, USA) were detected using an ultraviolet lamp (254 nm). Infrared (IR) and ^1^H-NMR spectra were taken by a Varian FT-IR spectrophotometer (Varian Medical Systems, Palo Alto, CA, USA) and a Varian AS 500 NMR spectrophotometer (Varian Medical Systems, Palo Alto, CA, USA), respectively. The chemical shift in the NMR spectra is presented in ppm, downfield from that of tetramethylsilane.

### 2.2. Synthesis of 1,1′-Sulfonylbis[4-(3-carboxy-4-hydroxyphenyl)azo]benzene (AS-DpS-AS)

DpS (248.0 mg) was dissolved in 10 mL of pre-chilled 5 M hydrochloric acid and sodium nitrite (NaNO_2_, 206.0 mg, 3 equiv.), and then was stirred for 1 h at 4 °C, followed by the addition of sulfamic acid (98.0 mg, 1 equiv.). SA (414.0 mg, 3 equiv.) dissolved in 10 mL of 1 M NaOH was added to the reaction solution, the pH was adjusted to approximately 9, and then reacted at 20–25 °C for 4 h. An appropriate volume of 1 M HCl was added to adjust the pH of the reaction mixture to approximately 5 and the precipitate formed during pH adjustment was isolated by centrifugation at 3000× *g* for 5 min. The precipitate was washed thrice with diethyl ether/acetone (1:1), followed by drying in a vacuum oven. AS-DpS-AS (M.W.: 546.51); Yield: 73%; mp: 263 °C (decomposition); IR (nujol mull), ν_max_ (cm^−1^): 1664 (C=O, -COOH in 5-ASA), 1147 and 1300 (S=O, O=S=O in DpS); ^1^H-NMR (DMSO-*d*_6_): δ = 8.34 (d, *J* = 9.8 Hz, 1H), 8.15 (dd, *J* = 8.9 Hz, 2H), 8.00 (dd, *J* = 8.6 Hz, 2H), 7.94 (dd, *J* = 19.1, 9.5 Hz, 1H), 6.94 (dd, *J* = 8.9 Hz, 1H).

### 2.3. High-Performance Liquid Chromatographic Analysis

The high-performance liquid chromatography (HPLC) system consisted of a Gilson model 306 pump, 151 variable UV detector, and model 234 autoinjector (Gilson, Middleton, WI, USA). Chromatographic separation was conducted using a symmetric C_18_ column (Hector, Theale, Berkshire, UK; 250 × 4.6 mm, 5 μm). Before injection for HPLC analysis, samples were filtered through membrane filters (0.45 μm, Revodix Inc., Gyeong-gi-do, Hanam, Korea). Mobile phases were prepared as follows: mobile phase A consisted of distilled water and acetonitrile (7:3, *v*/*v*), and mobile phase B consisted of acetonitrile and 1 mM phosphate buffer (pH 7.4) with 0.5 mM tetrabutylammonium chloride (1.5:8.5, *v*/*v*). The HPLC analysis was conducted at a flow rate of 1 mL/min. The eluate was monitored at 295 nm (for DpS), 367 nm (for AS-DpS-AS) and 330 nm (for 5-ASA) using a UV detector that measured the absorption with a sensitivity of AUFS 0.01. The retention times of DpS and AS-DpS-AS using mobile phase A were 9.8 min and 7.1 min, respectively, and that of 5-ASA using mobile phase B was 10.1 min.

### 2.4. Distribution Coefficient and Chemical Stability

Distribution coefficient of AS-DpS-AS was determined using water/1-octanol system as previously described [26]. The chemical stability of AS-DpS-AS was evaluated in pH 1.2 HCl-NaCl buffer and pH 6.8 isotonic phosphate buffer. AS-DpS-AS (0.1 mM) was incubated in the buffers overnight, and change in the concentration was monitored using HPLC analysis.

### 2.5. Animals

Seven-week-old male Sprague Dawley (SD) rats were purchased from Samtako Bio Korea (Gyeonggi-do, Osan, Korea) and housed in the animal care facility at Pusan National University, Busan, Korea, under controlled temperature, humidity, with a light/dark cycle (12 h/12 h). The animal protocol used in this study was reviewed and approved by the Pusan National University–Institutional Animal Care and Use Committee (PNU–IACUC) for ethical procedures and scientific care (Approval No: PNU-2021-3108, Approval data: 1 October 2021).

### 2.6. Analysis of Drug Concentration from Rat Intestinal Contents

Male SD rats (250–260 g) were euthanized with CO_2_ gas followed by a midline incision. The intestinal contents from the proximal small intestine (PSI), distal small intestine (DSI), and cecum were harvested and suspended in pH 6.8 isotonic phosphate buffer to prepare 20% (*w*/*v*) suspension. The cecal contents in the cecum were obtained under an N_2_ atmosphere in an atmospheric bag (AtmosBag, Sigma). Each suspension (5 mL) was mixed with AS-DpS-AS dissolved in 5 mL of pH 6.8 isotonic phosphate buffer (2.0 mM), followed by incubation at 37 °C (under nitrogen for the cecal contents). At the predetermined time intervals, a 0.5 mL portion of the mixture was transferred to a microtube and centrifuged at 10,000× *g* at 4 °C for 10 min. The supernatants (0.3 mL) were extracted with EA (0.3 mL), followed by centrifugation at 10,000× *g* at 4 °C for 7 min. The organic layer (0.2 mL) was transferred to a new microtube, which was evaporated and dissolved in mobile phase (0.2 mL), followed by filtration through a membrane filter (0.45 μm). The concentration of each drug in the filtrates (20 µL) was analyzed using HPLC. The same experiment was repeated (except for the use of an anaerobic bag) with small intestinal contents.

### 2.7. Analysis of Drug Concentration in Blood and Cecum of Rats

Male SD rats were allowed to starve for 24 h, except for drinking water. DpS (15 mg/kg, equivalent to 34 mg/kg AS-DpS-AS), AS-DpS-AS (34 mg/kg, equivalent to 50 mg/kg SSZ as 5-ASA), or SSZ (50 mg/kg) in PBS (1.0 mL) was administered to the rats by oral gavage. Blood samples were collected via cardiac puncture at appropriate intervals. To obtain the plasma, the blood samples were centrifuged at 10,000× *g* for 10 min at 4 °C. Plasma (0.25 mL) moved to new microtubes was extracted with EA (0.7 mL). The organic layer (0.5 mL) was evaporated and dissolved in a mobile phase (0.15 mL); this was filtered through a membrane filter (0.45 µm), and the filtrate (20.0 µL) was subjected to HPLC analysis. To determine concentrations of DpS and 5-ASA in the cecum, isotonic phosphate buffer (pH 6.8) was added to the cecal contents collected from the cecum of rats to prepare 10% suspension, which was centrifuged at 10,000× *g* for 10 min at 4 °C. For DpS analysis, the supernatant (0.2 mL) was extracted using EA (0.3 mL). The organic layer (0.2 mL) transferred to a new microtube was evaporated and dissolved in a mobile phase (0.2 mL). For analysis of 5-ASA, MeOH (0.9 mL) was added to the supernatant (0.1 mL) followed by centrifugation at 10,000× *g* for 10 min at 4 °C. Each solution was filtered through a membrane filter (0.45 μm), and the filtrate (20.0 µL) was used for HPLC analysis.

### 2.8. DNBS-Induced Rat Colitis

Experimental colitis was induced in rats as previously described [27]. Briefly, before the induction of colitis, male SD rats (250–260 g) were not fed for 24 h, except for drinking water. For induction of colitis, rats were anesthetized with isoflurane (Hana Pharm Co., LTD., Hwaseong, Korea) using the Small Animal O_2_ Single Flow Anesthesia System (LMS Co., LTD., Pyeongtaek, Korea), whose concentration was 3.5% for induction and 3% for maintenance with 1 L/min oxygen. Under adequate depth of anesthesia showing no response to physical stimuli, a rubber cannula (2 mm, OD) was inserted rectally to the splenic flexure of the colon, which was approximately 8 cm proximal to the anus. DNBS (48.0 mg) dissolved in 0.4 mL of 50% aqueous ethanol was instilled into the colon via a rubber cannula.

### 2.9. Evaluation of Anti-Colitic Effects

Three animal experiments were performed to study anti-colitic effects of drugs. One experiment was conducted to assess the anti-colitic effects of AS-DpS-AS and to compare them with those of SSZ. Rats were divided into five groups (n = 5 per group) and treated as follows: group 1 (normal group): oral gavage of 1.0 mL of PBS; group 2 (colitis group): oral gavage of 1.0 mL of PBS; group 3 (SSZ-treated colitis group): oral gavage of SSZ (50 mg/kg) in 1.0 mL of PBS; group 4 (AS-DpS-AS L-treated colitis group): oral gavage of AS-DpS-AS (17 mg/kg) in 1.0 mL of PBS; group 5 (AS-DpS-AS H-treated colitis group): oral gavage of AS-DpS-AS (34 mg/kg) in 1.0 mL of PBS. For the second experiment, the rats were divided into five groups (n = 5 per group) and treated as follows: group 1 (normal group): rectal administration of PBS (0.5 mL); group 2 (colitis group): rectal administration of PBS (0.5 mL); group 3 (5-ASA-treated colitis group): rectal administration of 5-ASA (20 mM) in 0.5 mL of PBS; group 4 (DpS-treated colitis group): rectal administration of DpS (10 mM) in 0.5 mL of PBS; group 5 (PMT-treated colitis group): rectal administration of a physical mixture (PMT) of DpS (10 mM) and 5-ASA (20 mM) in PBS (0.5 mL). For the third experiment, the rats were divided into three groups (n = 5 per group) and treated as follows: group 1 (normal group): oral gavage of 1.0 mL of PBS; group 2 (colitis group): oral gavage of 1.0 mL of PBS; group 3 (PMT-treated colitis group): oral gavage of PMT of DpS (15 mg/kg) and 5-ASA (19 mg/kg) in 1.0 mL of PBS. Three days after intracolonic instillation of DNBS, oral or rectal administration of each drug was conducted once per day for six days, and the anti-colitic effects were evaluated 24 h after the last treatment. Colonic damage score (CDS), representing colonic damage, was estimated by four independent observers blinded to the treatment conditions according to previously reported criteria [28]. The modified scoring system is as presented in Table S1, Supplementary Materials. Four independent observers blinded to the treatment conditions performed the CDS assessment. Myeloperoxidase (MPO) activity in the distal colon (4 cm) was measured as previously described [28]. One unit of MPO activity is defined as 1.0 μmol min^−1^ of peroxide degradation at 25 °C.

### 2.10. Western Blot Analysis

To prepare tissue lysates of the distal colon, tissue samples (0.2 g) were minced and homogenized in 2.0 mL of pre-chilled radioimmunoprecipitation assay (RIPA) buffer (50 mM Tris-HCl (pH 7.4), 1 mM EDTA, 0.7% Na deoxycholate, 1% NP-40, 150 mM NaCl, 0.3 μM aprotinin, 1 μM pepstatin, and 1 mM phenylmethylsulfonyl fluoride). After incubation on ice with frequent vortexing for 30 min, the homogenates were centrifuged at 10,000× *g* at 4 °C for 10 min and protein concentrations in the tissue lysates were determined using a bicinchoninic acid reagent (Thermo Fisher Scientific, Waltham, MA, USA) according to the manufacturer’s instructions. Tissue lysates were subjected to electrophoretic separation on 7.5% SDS-PAGE gel. Cyclooxygenase (COX)-2 and inducible nitric oxide synthase (iNOS) were detected using anti-COX-2 (sc-365374, Santa Cruz Biotechnology, Dallas, TX, USA) and anti-iNOS (NOS-2) (sc-7271, Santa Cruz Biotechnology) antibodies. SuperSignal chemiluminescence substrate (Thermo Fisher Scientific, Waltham, MA, USA) was used to visualize the bands. α-Tubulin (Santa Cruz Biotechnology) was used as the loading control. Western blot data were quantified using Image Lab software (version 5.2 build 14, Bio-Rad Laboratories, Inc., Hercules, CA, USA). The quantified results are presented as the mean of the quantified values for each Western blot in the figures (n = 5 for animal experiments).

### 2.11. ELISA for CINC-3

ELISA kits were used to determine levels of the inflammatory chemokine CINC-3 in the inflamed distal colon. The distal colons were minced in potassium phosphate buffer (pH 6.0), homogenized, then centrifuged at 10,000× *g* at 4 °C for 10 min. The lysates were subjected to ELISA performed according to the manufacturer’s instructions.

### 2.12. Data Analysis

The results are expressed as mean ± standard deviation (SD). One-way analysis of variance (ANOVA) followed by Tukey’s HSD test or Mann–Whitney *U* test (for CDS) was used to assess the differences between the groups. Differences were considered statistically significant at values of α or *p* < 0.05.

## 3. Results

### 3.1. Synthesis of DpS Azo-Linked with Two Molecules of 5-ASA (AS-DpS-AS)

**Figure 1:**
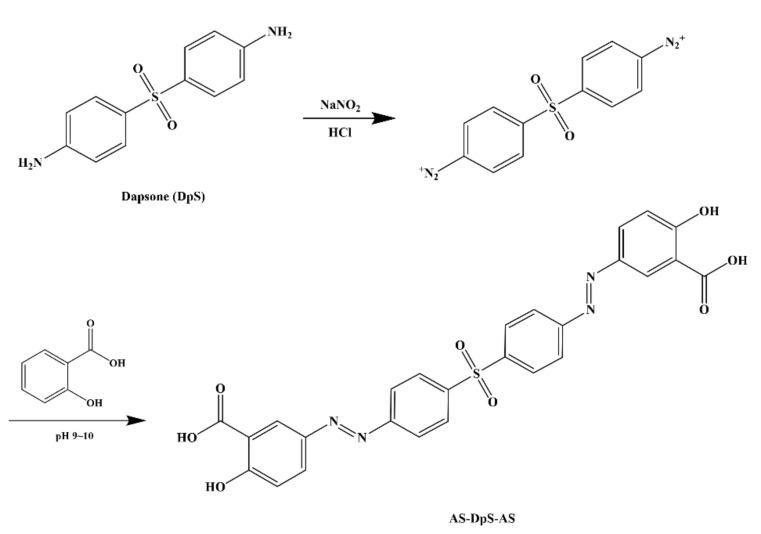
Synthesis of 1,1′-Sulfonylbis[4-(3-carboxy-4-hydroxyphenyl)azo]benzene (AS-DpS-AS).

DpS was azo-coupled with two molecules of 5-ASA to produce a colon-targeted prodrug, AS-DpS-AS. The synthesis of the azo compound was simple, as shown in Figure 1, and the formation of AS-DpS-AS was verified by IR and ^1^H-NMR. In the IR spectra of AS-DpS-AS, carbonyl stretching bands ascribed to the carboxylic acid group in 5-ASA were observed at 1664 cm^−1^, and the sulfone moiety in DpS was detected at 1300 cm^−1^. In the ^1^H-NMR spectra of AS-DpS-AS, aromatic proton signals originating from DpS and 5-ASA were detected with a slight downfield shift. The spectra are presented in Supplementary Figure S1.

### 3.2. AS-DpS-AS Is a Colon-Specific Prodrug Activated to DpS and Two Molecules of 5-ASA

**Figure 2:**
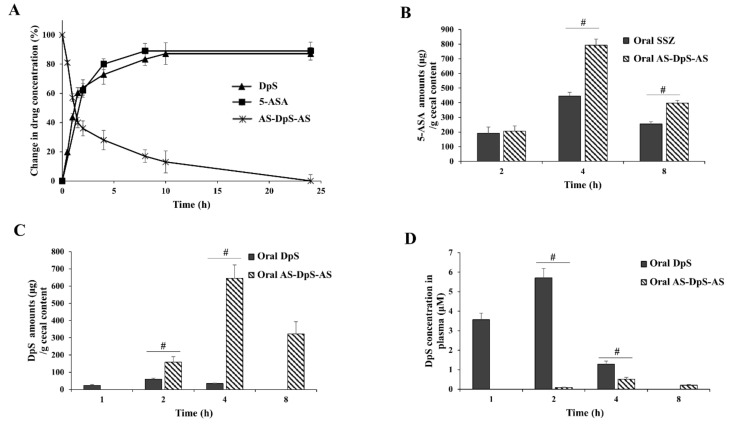
AS-DpS-AS is a colon-specific prodrug activated to DpS and two molecules of 5-ASA: (**A**) AS-DpS-AS (1.0 mM) were incubated in cecal contents suspended in pH 6.8 PBS (10%) under N_2_ atmosphere. The concentration of drugs was analyzed by HPLC at appropriate time intervals. The data represent mean ± SD (n = 3). Male SD rats (250–260 g) were allowed to fast for 24 h except for drinking water. (**B**) AS-DpS-AS (34 mg/kg, equivalent to 50 mg/kg of SSZ) or SSZ (50 mg/kg) suspended in pH 7.4 PBS was administered to rats via oral gavage. The rats were sacrificed at 2, 4, and 8 h after oral gavage, and concentrations of 5-ASA in the cecum were measured by HPLC. ^#^ *p* < 0.05. (**C**) DpS (15 mg/kg) or AS-DpS-AS (34 mg/kg, equivalent to 15 mg/kg of DpS) suspended in pH 7.4 PBS was administered to rats via oral gavage. The rats were sacrificed at 1, 2, and 4 h (for oral DpS) or 2, 4, and 8 h (for oral AS-DpS-AS) after oral gavage, and concentrations of DpS in the cecum were measured by HPLC. ^#^ *p* < 0.05. (**D**) Simultaneously, blood samples were obtained by cardiac puncture. DpS was analyzed in the blood by using HPLC. The data represent mean ± SD (n = 5). ^#^ *p* < 0.05.

In vitro and in vivo experiments were performed to examine the colon specificity of AS-DpS-AS. To test whether orally administered AS-DpS-AS (oral AS-DpS-AS) was delivered to the large intestine without significant loss in the upper GI tract, the distribution coefficient (log D_6.8_) of AS-DpS-AS, a parameter to estimate permeability across the gastrointestinal epithelial layer, was measured in a 1-octanol/isotonic phosphate buffer (pH 6.8) system. The value of log D_6.8_ of DpS (1.28) was decreased to 0.67 by azo-conjugation with 5-ASA. In addition, the chemical stability of AS-DpS-AS in buffers of pH 1.2 and 6.8, representing the pH of the stomach and small intestine, was examined. No change in the concentrations of AS-DpS-AS was observed for 10 h. For a compound to be a colon-specific prodrug, it must be converted to its parent drug after delivery to the large intestine. To test whether AS-DpS-AS satisfied this condition, AS-DpS-AS was incubated with small intestinal contents or with cecal contents in a glove box filled with nitrogen gas, and generation of parent drugs, 5-ASA and DpS, was monitored at the indicated time points. Although AS-DpS-AS remained stable in the small intestinal contents for up to 10 h, 5-ASA and DpS were detected during AS-DpS-AS disappearance; the concentration of 5-ASA was approximately twofold higher than that of DpS at each time point, as shown in Figure 2A. These in vitro results suggest that a large fraction of oral AS-DpS-AS was delivered to the large intestine, which was further converted to DpS and two molecules of 5-ASA. To verify this speculation, the concentration of 5-ASA was compared in the cecum of rats at 2, 4, and 8 h after oral administration of AS-DpS-AS and SSZ, a colon-specific prodrug of 5-ASA, at an equimolar dose of 5-ASA. In addition, to ensure the colon-specific delivery of DpS, the same experiment was conducted with DpS. As shown in Figure 2B, 5-ASA was detected in the cecum after oral administration of the prodrugs. The concentration of 5-ASA, including its maximal concentration, was higher with oral AS-DpS-AS, indicating that the colonic delivery efficiency of AS-DpS-AS was greater than that of SSZ. In parallel, as shown in Figure 2C, a much higher concentration of DpS was detected in the cecum with oral AS-DpS-AS than with oral DpS. Moreover, an approximately twofold concentration of 5-ASA (compared to the concentration of DpS) was detected at each time point, confirming colonic activation of AS-DpS-AS to DpS and two molecules of 5-ASA.

Since AS-DpS-AS at the dose used in the above experiment contained approximately nine times higher DpS amount than the usual adult dose of DpS (100 mg/day), we tested whether colon-specific delivery of DpS reduced the systemic absorption of DpS. The blood concentrations of DpS were compared at 2, 4, and 8 h (for oral AS-DpS-AS) and at 1, 2, and 4 h (for oral DpS) after oral administration of AS-DpS-AS and DpS at an equimolar dose. While oral DpS administration resulted in up to 5.6 μM of DpS in the blood, the maximal blood concentration of DpS was 0.5 μM after oral AS-DpS-AS administration (Figure 2D).

### 3.3. AS-DpS-AS Is More Effective against Rat Colitis than SSZ

**Figure 3:**
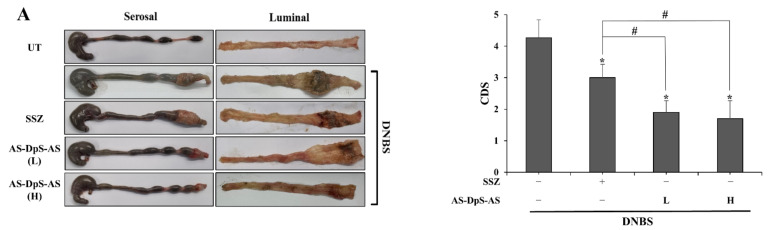

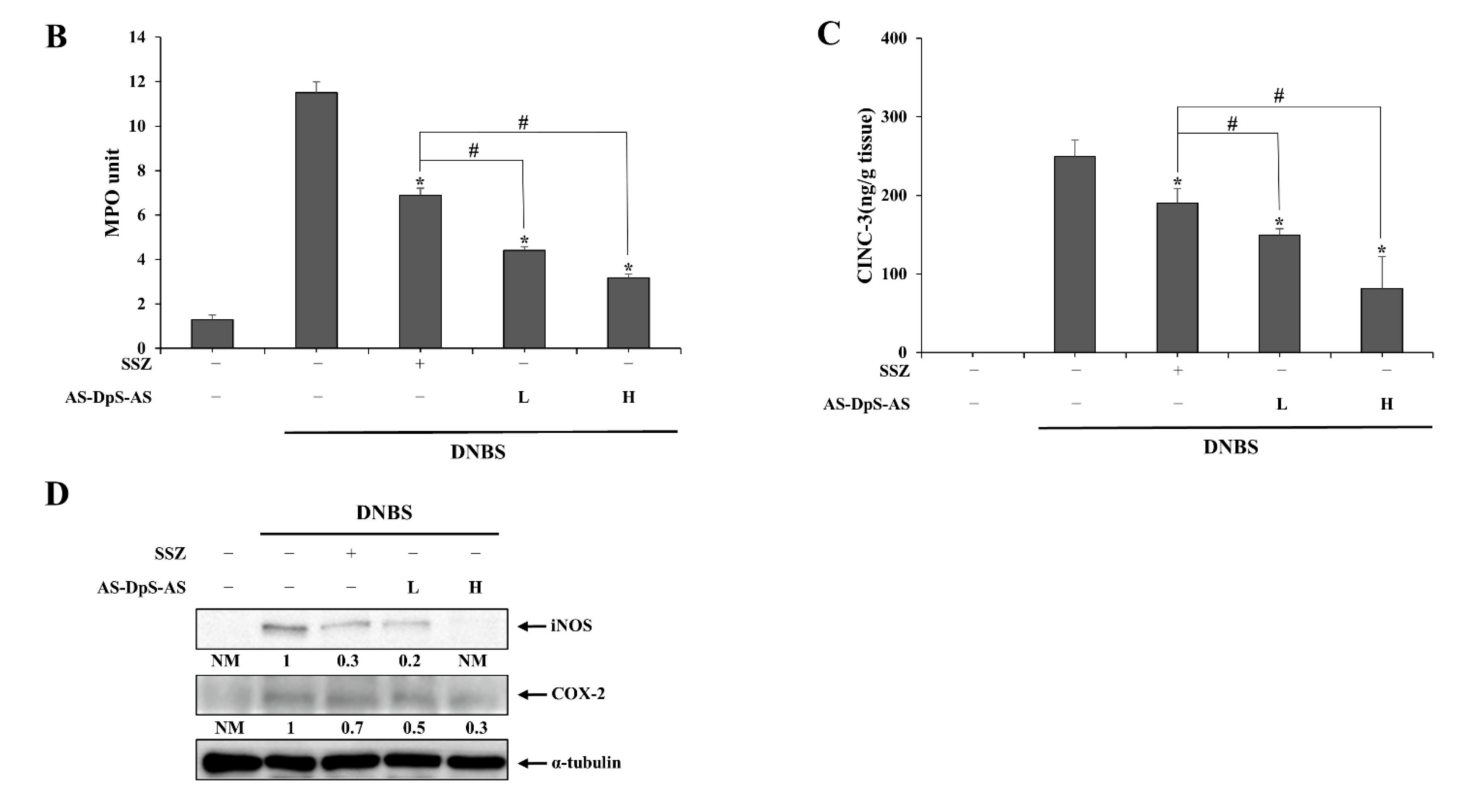
AS-DpS-AS is more effective against DNBS-induced rat colitis than SSZ. Three days after colitis induction by DNBS, SSZ (50 mg/kg) and AS-DpS-AS (L: 17 mg/kg, equivalent to 25 mg/kg of SSZ, H: 34 mg/kg, equivalent to 50 mg/kg of SSZ) were administered orally to rats once per day, and the rats were sacrificed after 6 days of treatment. (**A**) Left panel: Serosal and luminal sides of the distal colon of rats were photographed. Representative images are shown. Right panel: Colon damage score (CDS) was determined for each group, as described in the Section 2. * α < 0.05 vs. DNBS control, ^#^ α < 0.05. (**B**) Myeloperoxidase (MPO) activity was measured in the inflamed distal colon (4 cm). * *p* < 0.05 vs. control, ^#^ *p* < 0.05. (**C**) CINC-3 levels in the inflamed colon were measured using an ELISA kit. (**D**) iNOS and COX-2 levels were measured in the inflamed distal colon using Western blotting. * *p* < 0.05 vs. control, ^#^ *p* < 0.05. The data in (**A**–**C**) represent mean ± SD (*n* = 5). NM: not measurable.

To examine whether AS-DpS-AS elicited a higher therapeutic activity against rat colitis that can be used as a “me-better” alternative to SSZ, the anti-colitic effects of AS-DpS-AS were evaluated and compared with those of SSZ. Rats, in which colitis was induced by DNBS, were treated with two doses of oral AS-DpS-AS, once per day for 6 days. The doses of AS-DpS-AS were half-equivalent (low dose, L) and equivalent (high dose, H) to 50 mg/kg SSZ as 5-ASA. Simultaneously, the same experiment was repeated with SSZ (50 mg/kg). The rats were euthanized 24 h after the last treatment, and the anti-colitic effects of the drugs were assessed by evaluating the macroscopic and molecular indices in the inflamed distal colon. As shown in Figure 3A, rectal instillation of DNBS-induced severe colonic damage and inflammation in rats with hemorrhagic ulcers and scars, tissue edema, and luminal and serosal strictures. Oral AS-DpS-AS substantially mitigated the inflammatory damage presented by CDS and was more effective than oral SSZ. In parallel, oral AS-DpS-AS decreased MPO activity up to 38% (at low dose) and 29% (at high dose) of the DNBS control in the inflamed distal colon, whereas oral SSZ reduced MPO activity up to 60% of the DNBS control. In addition, levels of inflammatory mediators were determined in the inflamed distal colon. As shown in Figure 3C,D, oral AS-DpS-AS, which effectively lowered the levels of CINC-3 (Figure 3C), and COX-2 and iNOS (Figure 3D), were elevated in the inflamed distal colon of colitic rats. The anti-inflammatory effects of AS-DpS-AS were greater than those of SSZ. Except for COX-2 and iNOS, AS-DpS-AS, even at low doses, exhibited statistically significant anti-colitic effects compared to SSZ. To verify whether colon-targeted delivery was involved in the anti-colitic effects of AS-DpS-AS, the same animal experiment was conducted using a mixture of DpS and 5-ASA (equivalent to the high dose of AS-DpS-AS). Some anti-colitic effects were observed after oral gavage of a mixture of DpS and 5-ASA (Supplementary Figure S2). This finding was consistent with a recent study suggesting that DpS elicits anti-colitic activity in a mouse colitis model [17,21]. However, the effectiveness against colitis was very low compared to that obtained after oral introduction of AS-DpS-AS at the equimolar dose (Figure 2).

### 3.4. DpS and 5-ASA Effects Combine to Mitigate Colonic Damage and Inflammation in Rat Colitis

Our data showed that AS-DpS-AS is therapeutically superior to SSZ in rat colitis. Given that DpS and 5-ASA have anti-colitic activities, it is likely that 5-ASA and DpS (generated from AS-DpS-AS at the target site) combine to ameliorate rat colitis. To verify this hypothesis, we examined whether there was a combined action of these two agents against rat colitis upon rectal administration of DpS and/or 5-ASA.

**Figure 4:**
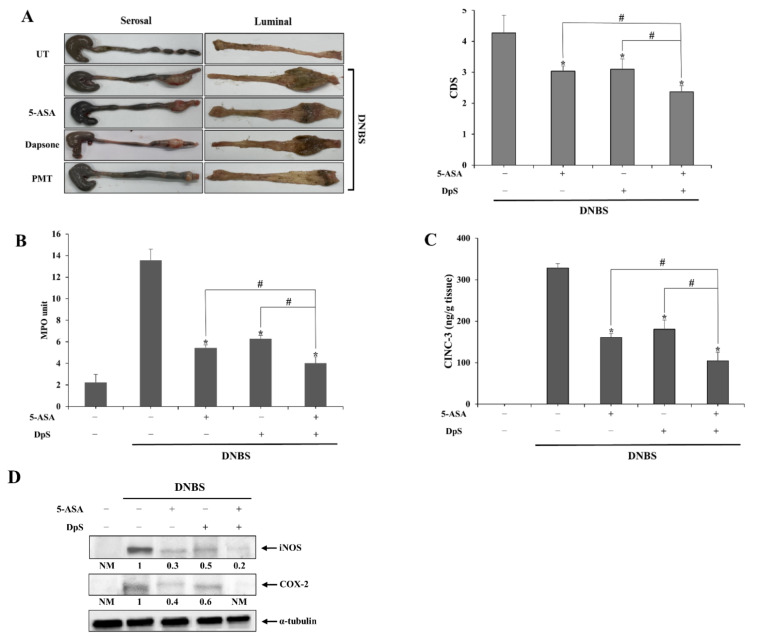
DpS and 5-ASA cooperate to alleviate rat colitis. Three days after colitis induction by DNBS, 5-ASA (19 mg/kg), DpS (15 mg/kg), and PMT were administered orally to rats once per day, and the rats were sacrificed after 6 days of treatment. (**A**) Left panel: Serosal and luminal sides of the distal colon of rats were photographed. Representative images are shown. Right panel: Colon damage score (CDS) was determined for each group, as described in the Section 2. * α < 0.05 vs. DNBS control, ^#^ α < 0.05. (**B**) Myeloperoxidase (MPO) activity was measured in the inflamed distal colon (4 cm). * *p* < 0.05 vs. control, ^#^ *p* < 0.05. (**C**) CINC-3 levels in the inflamed colon were measured using an ELISA kit. (**D**) iNOS and COX-2 levels were measured in the inflamed distal colon using Western blotting. * *p* < 0.05 vs. control, ^#^ *p* < 0.05. The data in (**A**–**C**) represent mean ± SD (n = 5). NM: not measurable.

Since the concentration of 5-ASA used for the treatment of rat colitis via the rectal route was 20–30 mM [29] and DpS was detected in the cecum at half the concentration of 5-ASA upon oral administration of As-DpS-AS, 20 mM of 5-ASA, and/or 10 mM of DpS were administered via the rectal route. As shown in Figure 4A, rectal DpS or rectal 5-ASA ameliorated colonic damage, and combined treatment with DpS and 5-ASA was more effective in preventing colonic damage than single treatments. Consistently, MPO activity in the inflamed distal colon was greatly reduced by rectal treatment with combination treatment, which was more effective than single treatments (Figure 4B). Consistent with the results of CDS and MPO activity, combined treatment, as well as a single treatment, decreased the levels of inflammatory mediators, CINC-3 (Figure 4C), COX-2, and iNOS (Figure 4D), where the suppressive effects of combined treatment were significantly superior to those of the single treatments.

## 4. Discussion

SSZ is a mesalazine-based prodrug used to treat IBD. Owing to the disadvantages of SSZ, such as side effects and low anti-inflammatory efficacy, efforts have been made to develop a “me-better” (or “me-too”) drug for SSZ. These efforts led to the development of olsalazine and balsalazide, which have improved toxicological properties [7]. To improve anti-inflammatory activity as well as toxicological properties, DpS azo-conjugated with 5-ASA, AS-DpS-AS, was designed and evaluated as an alternative to SSZ.

Our data demonstrated that AS-DpS-AS acts as a colon-specific prodrug that produces DpS and two molecules of 5-ASA. Oral AS-DpS-AS delivered a greater amount of 5-ASA to the cecum than oral SSZ upon administration of an equivalent dose of 5-ASA, indicating better colonic delivery efficiency of AS-DpS-AS. This is ascribed largely to the structural features of AS-DpS-AS, having two carboxylic groups (unlike SSZ with one carboxylic group), resulting in a lower distribution coefficient, a parameter predicting permeability through the epithelial layer of the GI tract. In addition, AS-DpS-AS was cleaved into DpS and two 5-ASA molecules in the large intestine. Stoichiometric release of 5-ASA and DpS was observed after incubation of AS-DpS-AS in the cecal contents (Figure 2A) and was reproduced in the cecum upon oral administration of AS-DpS-AS (Figure 2B,C). In agreement with the general features of colon-specific drug delivery [10], AS-DpS-AS limited the systemic absorption of DpS while supplying a large amount of DpS in the large intestine, likely potentiating therapeutic activity and preventing systemic side effects. Comparison of the plasma concentrations of DpS after oral AS-DpS-AS and DpS clearly showed much lower plasma concentrations of DpS with oral AS-DpS-AS. Additionally, a much greater amount of DpS was detected in the cecum with oral AS-DpS-AS than with oral DpS, as shown in Figure 2C. These results suggest that while a large fraction of oral DpS is absorbed systemically during transit through the upper intestine, a large fraction of oral AS-DpS-AS arrives intact in the large intestine and is converted to DpS and 5-ASA.

In addition to the colonic delivery efficiency, AS-DpS-AS is therapeutically superior to SSZ in treating experimental colitis. Both oral AS-DpS-AS and SSZ significantly ameliorated colonic damage and inflammation, and AS-DpS-AS was more effective against rat colitis than SSZ. Even at a low dose of AS-DpS-AS corresponding to a half-equimolar dose of SSZ, AS-DpS-AS was significantly superior to SSZ in improving most colitis indices, except for the molecular indices COX-2 and iNOS. Our data showed that oral PMT (physical mixture of 5-ASA + DpS) elicited weaker anti-colitic activity than AS-DpS-AS, suggesting that colon-specific delivery is associated with the anti-colitic activity of AS-DpS-AS.

Consistent with our hypothesis based on the anti-colitic activity of DpS [20,21], AS-DpS-AS was found to be more effective against colitis than SSZ. These results suggest that AS-DpS-AS acts as a mutual colon-specific prodrug via the cooperative anti-colitic action of DpS and 5-ASA. This argument was strongly supported by data showing that combined intracolonic treatment with DpS and 5-ASA was more effective in ameliorating colonic damage and inflammation than single treatment with either 5-ASA or DpS.

Unlike sulfapyridine, the carrier of SSZ, DpS, has been used to treat leprosy, requiring daily treatment for 1–2 years [17]. Therefore, DpS is likely to be more tolerable for long-term use. Although AS-DpS-AS at high dose comprises a ninefold dose of the usual adult dose of DpS (100 mg/day) [17], this may not increase the risk of side effects, considering the substantial reduction in systemic absorption of DpS upon oral AS-DpS-AS. Figure 2D showed that the maximal plasma concentration of DpS was reduced from 5.6 μM (after oral DpS) to 0.5 μM (after oral AS-DpS-AS) upon oral administration of the drugs at an equimolar dose.

One of the practical issues in using SSZ in clinics is poor patient compliance due to its high dose [30]. Our data indicated that AS-DpS-AS could improve patient compliance. AS-DpS-AS has a structural feature that contains two molecules of 5-ASA, thereby possessing the intrinsic potential to reduce the dose of AS-DpS-AS, even at a dose equimolar to SSZ as 5-ASA. Moreover, our data showed that AS-DpS-AS was more effective against rat colitis than SSZ, which seems to be valid even at a low dose of AS-DpS-AS corresponding to a half-equimolar dose of 5-ASA, suggesting that the effective dose of AS-DpS-AS against IBD could be three times less than that of SSZ.

## 5. Conclusions

Our cumulative results suggest that AS-DpS-AS may be a “me-better” drug of SSZ as it can improve therapeutic and toxicological properties, as well as patient compliance.

## Data Availability

The data presented in this study are available in article or Supplementary Materials here.

## Supplementary Materials

The following supporting information can be downloaded at: https://www.mdpi.com/article/10.3390/pharmaceutics14030684/s1. Table S1: Modified scoring system for assessment of CDS, Figure S1: IR and ^1^H-NMR spectra of AS-DpS-AS, and Figure S2: Anti-colitic effects of oral PMT against DNBS-induced rat colitis.
