# Supplementary material for: Dapsone Azo-Linked with Two Mesalazine Moieties Is a “Me-Better” Alternative to Sulfasalazine
Source: Pharmaceutics. 2022 Mar 21;14(3):684. doi: 10.3390/pharmaceutics14030684 (PMC8949065; doi:10.3390/pharmaceutics14030684)
Supplement: Supplementary file 1 [file pharmaceutics-14-00684-s001.zip › pharmaceutics-1646457-supplementary.pdf]

pharmaceutics

MDPI

Supplementary Materials: Dapsone Azo-Linked with Two Mesalazine Moieties Is a "Me-Better" Alternative to Sulfasalazine

Changyu Kang, Jaejeong Kim, Sanghyun Ju, Sohee Park, Jin-Wook Yoo, In-Soo Yoon, Min-Soo Kim and Yunjin Jung

Table S1. IR and ^{1}H-NMR spectra of AS-DpS-AS.

Score	Feature
0	normal appearance
1	localized hyperemia but no ulcer
2	linear ulcers without significant inflammation
3	2–4 cm site of inflammation and ulceration
4	serosal adhesion to other organs, 2–4 cm site of inflammation and ulceration
5	stricture, serosal adhesion involving several bowel loops, <4 cm site of inflammation and ulceration

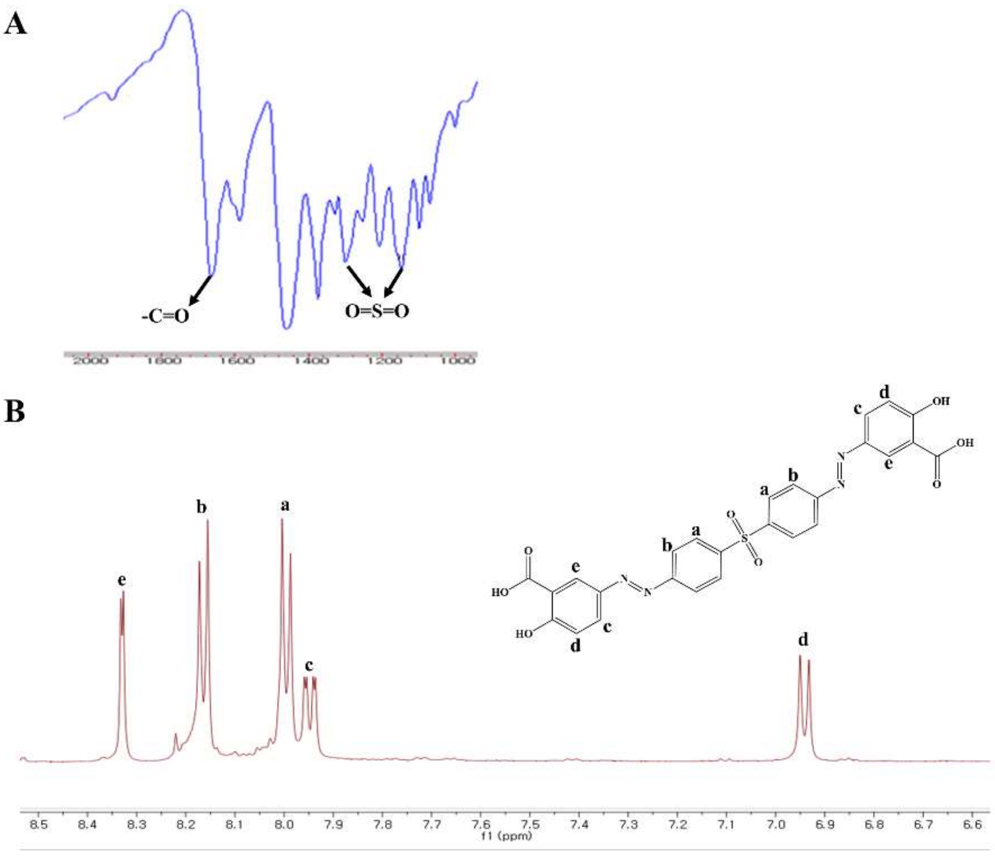

Figure S1. IR and ^{1}H-NMR spectra of AS-DpS-AS. (**A**) IR spectrum of AS-DpS-AS, (**B**) ^{1}H-NMR spectrum of AS-DpS-AS.

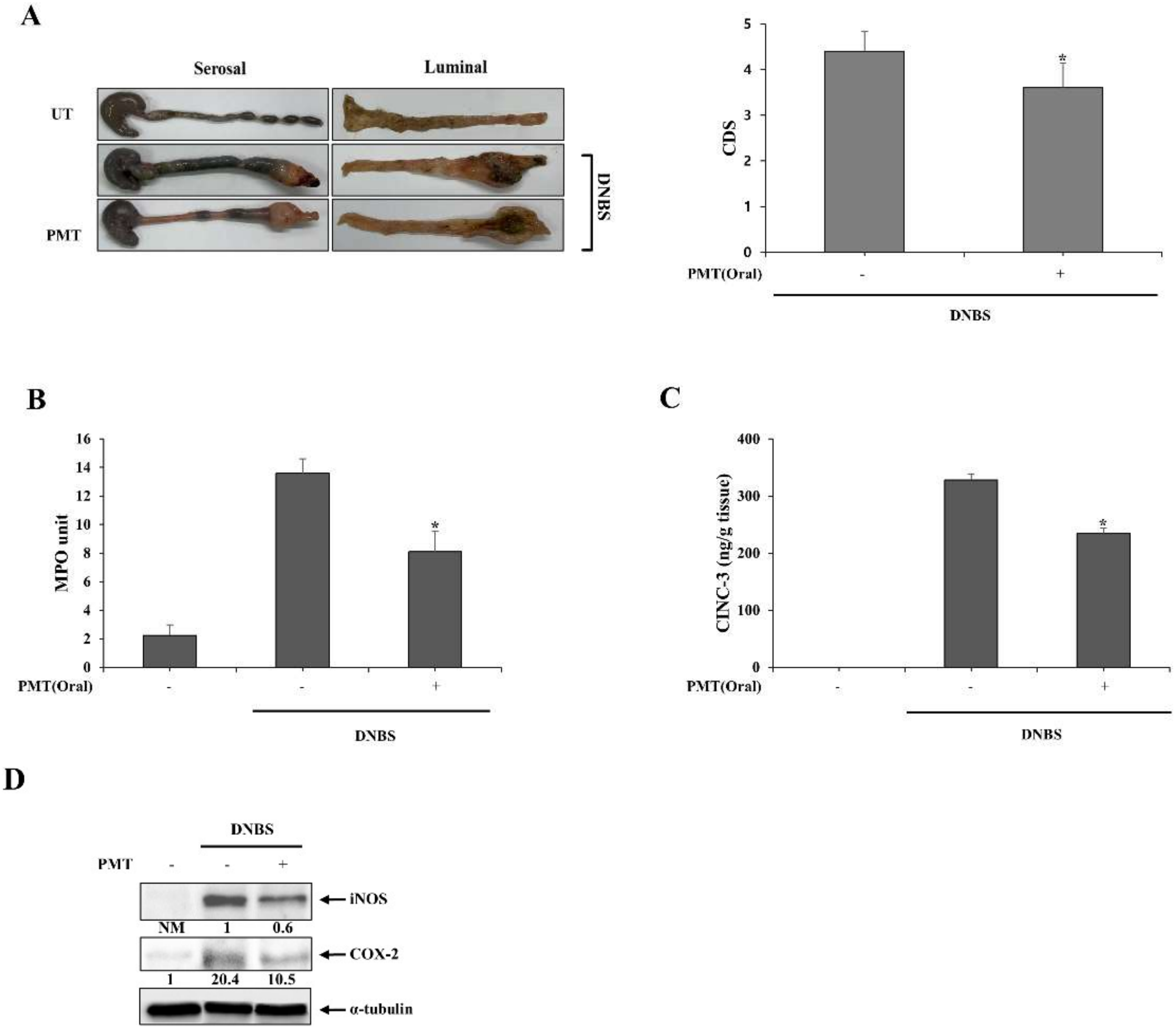

Figure S2. Anti-colitic effects of oral PMT against DNBS-induced rat colitis. Three days after colitis induction by DNBS, a physical mixture (PMT) of 5-ASA (19 mg/kg) and DpS (15 mg/kg) was administered orally to rats once per day, and the rats were sacrificed after 6 days of treatment. (A) Left panel: Serosal and luminal sides of the distal colon of rats were photographed. Representative images are shown. Right panel: Colon damage score (CDS) was determined for each group, as described in the Methods section. $^{*}\alpha < 0.05$, vs. the DNBS control. (B) Myeloperoxidase (MPO) activity was measured in the inflamed distal colon (4 cm). (C) CINC-3 levels in the inflamed colon were measured using an ELISA kit. (D) iNOS and COX-2 levels were measured in the inflamed distal colon using western blotting. $^{*}P < 0.05$ vs. control. The data in A, B, and C represent mean ± SD (n = 5).
